# Extensively drug-resistant (XDR) *Neisseria gonorrhoeae* causing possible gonorrhoea treatment failure with ceftriaxone plus azithromycin in Austria, April 2022

**DOI:** 10.2807/1560-7917.ES.2022.27.24.2200455

**Published:** 2022-06-16

**Authors:** Sonja Pleininger, Alexander Indra, Daniel Golparian, Florian Heger, Stefanie Schindler, Susanne Jacobsson, Stefan Heidler, Magnus Unemo

**Affiliations:** 1 Austrian Agency for Health and Food Safety, Vienna, Austria; 2 WHO Collaborating Centre for Gonorrhoea and Other STIs, National Reference Laboratory for STIs, Faculty of Medicine and Health, Örebro University, Örebro, Sweden; 3 Urology Department, LKH Hochsteiermark, Leoben, Austria; 4 Institute for Global Health, University College London, London, United Kingdom

**Keywords:** *Neisseria gonorrhoeae*, extensively drug-resistant, XDR, ceftriaxone resistance, high-level azithromycin resistance

## Abstract

We describe a gonorrhoea case with ceftriaxone plus high-level azithromycin resistance. In April 2022, an Austrian heterosexual male was diagnosed with gonorrhoea after sexual intercourse with a female sex worker in Cambodia. Recommended treatment with ceftriaxone (1 g) plus azithromycin (1.5 g) possibly failed. Worryingly, this is the second strain in an Asian *Neisseria gonorrhoeae* genomic sublineage including high-level azithromycin-resistant strains that developed ceftriaxone resistance by acquisition of mosaic *penA*-60.001. Enhanced resistance surveillance and actions are imperative to prevent spread.

Multidrug- and extensively drug-resistant (XDR) *Neisseria gonorrhoeae* strains are major global public health concerns. In most countries worldwide, ceftriaxone (0.25–1 g) as monotherapy or combined with azithromycin (1–2 g) are recommended first-line gonorrhoea therapies, and resistance to ceftriaxone and azithromycin hampers therapy with these two last remaining treatment options [1-3]. We describe the second global XDR *N. gonorrhoeae* strain [4,5], with high-level resistance to azithromycin and resistance to ceftriaxone, cefixime, cefotaxime, ciprofloxacin and tetracycline, which caused a possible gonorrhoea treatment failure with ceftriaxone (1 g) plus azithromycin (1.5 g) in a case from Austria who was infected after condomless sexual contact in Cambodia.

## Clinical case description and microbiology

In April 2022, a heterosexual male patient in his 50s consulted a urology department in Austria because of painful urination and urethral discharge. Five days before onset of symptoms, he had condomless heterosexual contact with a female commercial sex worker in Cambodia, who could not be traced. Based on sexual history, a urethral swab was taken, and *N. gonorrhoeae* culture (AT159 strain) verified the gonorrhoea diagnosis. The patient was treated with ceftriaxone (1 g intramuscularly) plus azithromycin (1.5 g single oral dose), according to European recommendations [1] but using a slightly adapted azithromycin dosing (1.5 g instead of 2 g). Approximately 2 weeks later at a follow-up visit, symptoms had resolved. Test of cure using culture of urethral, rectal and pharyngeal samples was negative, however, a PCR test (Allinity, Abbott, Chicago, Illinois, United States (US)) from the urethral swab culture sample was *N. gonorrhoeae*-positive. Because no post-treatment gonococcal isolates were available, the case was considered as a possible treatment failure [4,5]. Based on the antimicrobial susceptibility testing results (Table), additional treatment with amoxicillin-clavulanic acid (1 g) twice a day for 7 days was prescribed. At the test of cure after this second treatment, a urethral sample was *N. gonorrhoeae* culture-negative; unfortunately, no urine PCR sample was available. The patient was negative in *Chlamydia trachomatis* PCR (FluoroType CT, Bruker, Billerica, US) and *Mycoplasma genitalium* testing (MYCOPLASMA IST 3, bioMerieux SA, Marcy l’Etoile, France). The patient did not consent to HIV and syphilis testing.

**Table:** Antimicrobial minimum inhibitory concentration of the extensively drug-resistant *Neisseria gonorrhoeae* strain (AT159) causing a possible gonorrhoea treatment failure, Austria^a^, April 2022 (n = 1 strain)

Antimicrobial	MIC in mg/L	Interpretation (EUCAST v 12.0 [6])
Ceftriaxone	0.25	Resistant
Cefixime	1	Resistant
Cefotaxime	0.5	Resistant
Azithromycin	> 256	High-level resistant
Ciprofloxacin	16	Resistant
Tetracycline	16	Resistant
Penicillin G	0.5	Susceptible, increased exposure
Spectinomycin	16	Susceptible
Gentamicin	4	NA (Wild-type MIC)
Rifampicin	0.125	NA (Wild-type MIC)
Ertapenem	0.016	NA (Wild-type MIC)
Zoliflodacin^b^	0.032	NA (Wild-type MIC)
Lefamulin	0.125	NA (Wild-type MIC)

EUCAST: European Committee on Antimicrobial Susceptibility Testing; MIC: minimum inhibitory concentration; NA: not applicable (due to lack of interpretative breakpoints).

^a^ The patient reported having had sexual intercourse with a female sex worker in Cambodia.

^b^ Pre-licensing international phase III randomised clinical trial is ongoing.

### Antimicrobial susceptibility testing

Minimum inhibitory concentrations (MICs) of antimicrobials were determined at the National Reference Laboratory for Gonorrhoea in Vienna, Austria and the WHO Collaborating Centre for Gonorrhoea and Other STIs in Örebro, Sweden by Etest (bioMerieux SA, Marcy l’Etoile, France), in accordance with the manufacturer’s instruction, or agar dilution (zoliflodacin and lefamulin). The MICs of 13 antimicrobials and, where available, interpretations using breakpoints according to European Committee on Antimicrobial Susceptibility Testing (EUCAST) version 12.0 [6] are shown in the Table.

Briefly, AT159 showed high-level resistance to azithromycin (MIC > 256 mg/L), and resistance to ceftriaxone, cefixime, cefotaxime, ciprofloxacin, and tetracycline (Table). Accordingly, AT159 was XDR, according to international definitions [4,5]. However, the strain was susceptible to spectinomycin, ‘intermediate susceptible’ (i.e. susceptible, increased exposure) to benzylpenicillin and showed wild-type MICs of gentamicin, rifampicin, ertapenem and the new antimicrobials zoliflodacin and lefamulin (Table). AT159 did not produce beta-lactamase, according to a nitrocefin test (Becton Dickinson, Franklin Lakes, New Jersey, US). Only one gonococcal strain with ceftriaxone resistance combined with high-level azithromycin resistance (subsequently assigned as the WHO Q reference strain) has been previously described globally, which caused three gonorrhoea cases in the United Kingdom (UK) and Australia in 2018. These cases also had links to South East Asia [7,8].

### Molecular investigation

The isolate AT159 was sequenced with NextSeq 550 (Illumina, San Diego, CA, US) and sequencing reads submitted to the European Nucleotide Archive (accession number PRJEB53054). Quality control, assembly and characterisation of molecular epidemiological sequence types (STs) and antimicrobial resistance determinants [9,10] were performed using a customised CLC Genomics Workbench (Qiagen), as previously described [11,12]. All sequences were also assembled using Spades and uploaded to the Pathogenwatch platform [9] for core genome multilocus sequence typing (cgMLST).

Briefly, AT159 was assigned to the novel MLST ST16406 and *N. gonorrhoeae* sequence typing for antimicrobial resistance (NG-STAR) type ST4465. The high-level azithromycin resistance was caused by the 23S rRNA A2059G target mutation (in all four 23S rRNA gene alleles) and the resistance to the extended-spectrum cephalosporins (ceftriaxone, cefixime and cefotaxime) by the mosaic *penA*-60.001 allele [7-10,13]. This mosaic *penA*-60.001 allele is also causing ceftriaxone resistance in the internationally spreading FC428 strain, first reported in Japan in 2015 [13] and subsequently identified in many countries globally including in Europe [14-18], although FC428 and AT159 are genomically widely different (data not shown). However, phylogenomic analysis of the draft AT159 genome sequence and the most closely related gonococcal genomes from several Asian countries [12], where many ceftriaxone-resistant strains appear to have emerged [7,8,13-18], showed that AT159 belongs to the same sublineage as WHO Q [7,8] (Figure), but differs by 313 alleles.

**Figure:**
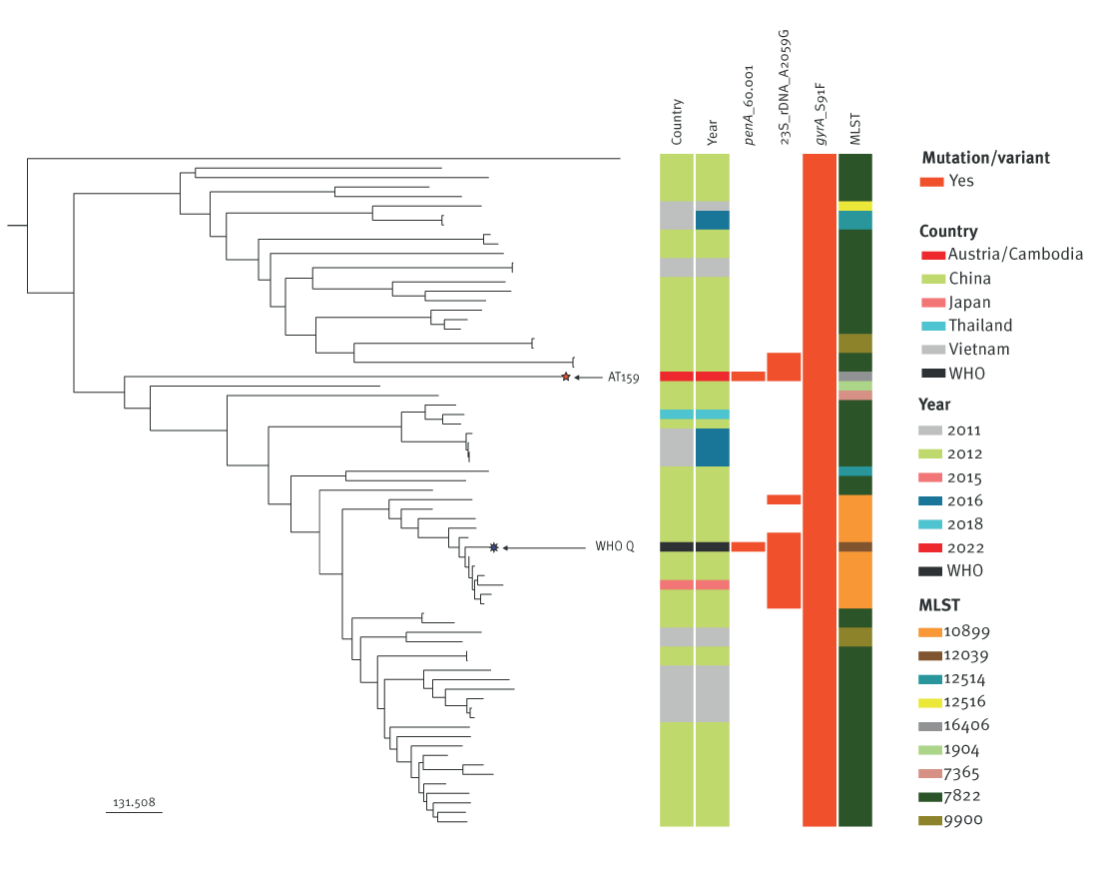
Phylogeny of the most closely related *Neisseria gonorrhoeae* genome sequences from a recent study [12], Asia, 2011–2018 to the extensively drug-resistant *N. gonorrhoeae* strain (AT159) causing a possible gonorrhoea treatment failure, Austria^a^, April 2022 (n = 71 genome sequences)

MLST: Multilocus sequence typing; UK: United Kingdom; WHO: World Health Organization.

^a^ The patient reported having had sexual intercourse with a female sex worker in Cambodia.

WHO Q (purple star) was identified in the UK, but the infection was in Thailand [7]. Mutations or alleles conferring resistance to ceftriaxone (*penA* 60.001), azithromycin (23S rDNA A2059G) and ciprofloxacin (*gyrA* S91F) [9,10] are shown in red.

Genome assemblies of closely related Asian isolates (n = 70) [12] to AT159 (orange star) were uploaded and analysed on the Pathogenwatch platform [9,19] including a phylogenetic inference. Briefly, Pathogenwatch [9] provides a cgMLST defined by the 2016 *Neisseria* *gonorrhoeae* reference strains [20] and each assembly is linked to the closest reference by comparing the substitution score profiles to the reference core profiles excluding indels and non-ACTG characters. cgMLST profiles are then clustered by calculating distances between each assembly which shares a given cgMLST scheme. These distances are calculated as the number of different loci for the scheme, ignoring any missing loci. A clustering is performed using Single Linkage Clustering based on these calculated pairwise distances [9], see https://cgps.gitbook.io/pathogenwatch/technical-descriptions/cgmlst-clusters. A tree is finally constructed by writing all scaled pairwise scores to a matrix, and running a neighbour-joining implementation to construct a tree that is finally midpoint rooted [9], see https://cgps.gitbook.io/pathogenwatch/technical-descriptions/core-genome-tree.

Concern is warranted given that many strains in this Asian *N.* *gonorrhoeae* sublineage show a high-level azithromycin resistance (because of 23S rRNA A2059G mutations) and that some of these strains, such as AT159 and WHO Q [7,8], appear to additionally develop ceftriaxone resistance by acquisition of mosaic *penA*-60.001 (Figure), most likely through transformation. All the strains in this sublineage are additionally resistant to ciprofloxacin (Figure).

## Discussion

We describe the second global XDR gonococcal strain [4,5], with high-level resistance to azithromycin and resistance to ceftriaxone, cefixime, cefotaxime, ciprofloxacin, and tetracycline, which caused a possible gonorrhoea treatment failure with recommended ceftriaxone plus azithromycin therapy. The case from Austria reported about condomless sexual contact with a female sex worker in Cambodia 5 days before onset of symptoms. A limitation of our study is that the female sex worker could not be traced and, thus, no gonococcal isolate or other samples from this female were available to link to AT159. Notably, in 2019, another ceftriaxone-resistant gonococcal strain was reported in France, also after stating sexual contact with a female in Cambodia [21]. However, this strain belonged to the internationally spreading FC428 clone [13-18,21].

In the absence of a gonococcal vaccine, early and effective diagnosis and antimicrobial treatment of gonorrhoea are essential [1,3,10]. However, *N. gonorrhoeae* has developed resistance to all classes of antimicrobials since introduction of antimicrobial treatment in the 1930s [1,7,8,10,13-18,21,22]. XDR *N. gonorrhoeae* strains, including those with resistance to all available treatment options, are a major global public health concern. They pose a risk of treatment failure and serious complications/sequelae on the individual level but also compromise the management and control of gonorrhoea on the public health level. Resistance or decreased susceptibility to ceftriaxone and azithromycin resistance in *N. gonorrhoeae* has been reported worldwide [22]. In recent years in Europe, the susceptibility to ceftriaxone has increased but, worryingly, the resistance to azithromycin rapidly increased [19,23]. Furthermore, sporadic ceftriaxone-resistant strains have been identified in several European countries [1,5,7,15,18,19,22,23]. However, the XDR strain described in the present paper is the second global gonococcal strain with ceftriaxone resistance combined with high-level azithromycin resistance, with relatively close relationship with WHO Q [7,8], although not the same subvariant (Figure). It is of concern that high-level azithromycin-resistant strains in an Asian *N.* *gonorrhoeae* genomic sublineage are able to develop ceftriaxone resistance by acquisition of mosaic *penA*-60.001. If such strains manage to establish a sustained transmission, many gonorrhoea cases might become untreatable. Promisingly, the XDR AT159 strain had wild-type MICs of the novel gonorrhoea antimicrobials lefamulin and zoliflodacin, which is in a phase 3 randomised clinical trial [10,12,19,22].

## Conclusions

Ceftriaxone resistance combined with high-level azithromycin resistance in *N. gonorrhoeae* is a concern for future treatment of gonorrhoea and poses a major global public health threat. Improved prevention (including condom use), early and accurate diagnosis and effective, affordable and accessible treatment (ideally including test of cure and contact notification and treatment) of gonorrhoea are imperative. Furthermore, increased awareness of the spread of ceftriaxone-resistant strains and rapid identification and eradication of the sporadic XDR gonococcal strains are essential to avoid any clonal expansion and sustained transmission of these strains. Enhanced antimicrobial resistance surveillance, ideally including test of cure and whole-genome sequencing, nationally and internationally, particularly in Asia where many ceftriaxone-resistant strains appear to have emerged [5,7,8,13-18,21,22], is of highest importance. Ultimately, novel antimicrobials for effective treatment of gonorrhoea and/or a sufficiently effective gonococcal vaccine will be crucial.
